# Emotional Context Influences Micro-Expression Recognition

**DOI:** 10.1371/journal.pone.0095018

**Published:** 2014-04-15

**Authors:** Ming Zhang, Qiufang Fu, Yu-Hsin Chen, Xiaolan Fu

**Affiliations:** 1 State Key Laboratory of Brain and Cognitive Science, Institute of Psychology, Chinese Academy of Sciences, Beijing, China; 2 University of Chinese Academy of Sciences, Beijing, China; University of Akron, United States of America

## Abstract

Micro-expressions are often embedded in a flow of expressions including both neutral and other facial expressions. However, it remains unclear whether the types of facial expressions appearing before and after the micro-expression, i.e., the emotional context, influence micro-expression recognition. To address this question, the present study used a modified METT (Micro-Expression Training Tool) paradigm that required participants to recognize the target micro-expressions presented briefly between two identical emotional faces. The results of Experiments 1 and 2 showed that negative context impaired the recognition of micro-expressions regardless of the duration of the target micro-expression. Stimulus-difference between the context and target micro-expression was accounted for in Experiment 3. Results showed that a context effect on micro-expression recognition persists even when the stimulus similarity between the context and target micro-expressions was controlled. Therefore, our results not only provided evidence for the context effect on micro-expression recognition but also suggested that the context effect might result from both the stimulus and valence differences.

## Introduction

Micro-expressions are extremely quick facial expressions [1] that usually last for 1/25 s to 1/5 s [2]. Like facial expressions, micro-expressions also include some basic emotions [3], [4], such as anger and fear [5]. Normally, a micro-expression is embedded in the flow of expressions and occurs when people try to conceal or repress their emotions [6]. Previous research suggests that micro-expressions are important cues for revealing true feelings and detecting deceptive behaviors [7]. However, people usually have difficulties detecting or recognizing micro-expressions [8].

**Figure 1:**
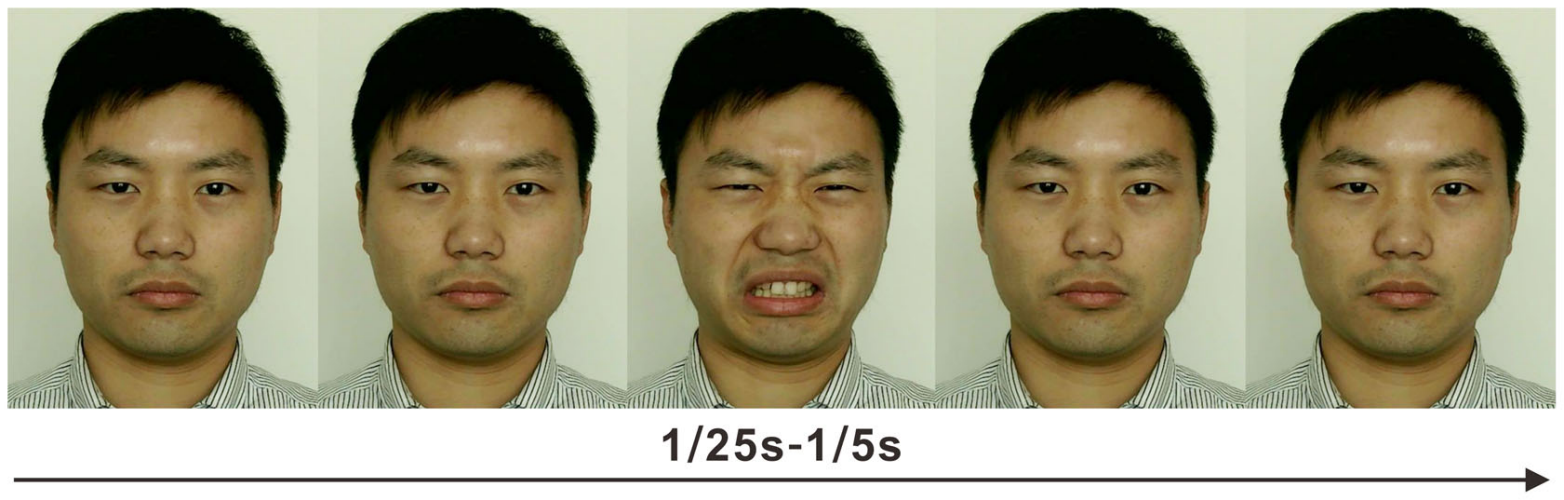
A disgust micro-expression occurred between facial expressions (created according to METT [10]).

Synthesized micro-expressions refer to artificially created micro-expressions in which an emotional expression is inserted between two neutral expressions (See Figure 1). Synthesized micro-expressions are commonly used in micro-expression recognition research as well as training materials [9], such as those in Ekman’s micro-expression training tool (METT), which aimed to improve people’s ability to recognize expressions [10]. In addition, synthesized micro-expressions are also employed to investigate the characteristics and other influencing factors in micro-expression recognition research [11], [12]. For example, Shen et al. (2012), using the neutral-emotional-neutral paradigm, found that recognition accuracy rates gradually increased as presentation duration became longer but was within 200 ms. Although previous studies have employed neutral expressions before and after the emotional expression, research has indicated that micro-expressions may be embedded not only in neutral expressions but also in other facial expressions, such as happiness and sadness [13]. To date, it remains unknown whether the recognition of micro-expressions is influenced by the types of facial expressions appearing before and after the micro-expression, i.e., the emotional context.

Previous studies have demonstrated that the current context influences recognition of facial expressions [14]. For example, negative facial expressions are recognized more quickly and accurately with negative context than with positive context [15]. Moreover, it has also been shown that the emotional valence information appearing before the facial expression influences facial expression recognition [16], [17]. Some affective priming studies have found that the primes have different roles in the recognition of the different types of facial expressions [18], [19]. For example, anger expressions are recognized more quickly and accurately when the prime is an angry face than when it is a happy face [20]. It has also been observed that happy faces are recognized more accurately after positive primes than after negative ones, whereas sad expressions are recognized more accurately after negative primes [21]. The effects of emotional context on facial expression recognition have also been observed and confirmed in numerous cognitive neuroscience research (See [22], [23] for a detailed overview).

In priming tasks, the prime is often presented for a short duration and the target for a long duration, whereas the reverse is employed in synthesized micro-expression tasks. Both priming and synthesized micro-expression tasks, however, involve the processing of previous emotional stimuli and target stimuli. Primes presented for long durations may lead to greater priming effect; moreover, emotional information has been observed to influence attention [24] according to the emotional regulation theory [25]. These findings lead us to predict that micro-expression recognition would be influenced by emotional context. The purpose of the current study was to investigate whether emotional context plays a role in micro-expression recognition. To achieve this goal, we extended upon the METT [10] design to determine whether emotional context influences micro-expression recognition. Furthermore, we also assessed the underlying factors (i.e. valence difference and stimuli difference) that may lead to the context effect on micro-expression recognition.

## Experiment 1

Experiment 1 was designed to investigate whether there was an effect of emotional context on micro-expressions recognition. We chose the facial expressions of anger, disgust, happiness, fear, and surprise as the five target micro-expressions and the facial expressions of sad, neutral, and happy as the three context expressions in this experiment. We hypothesized that the recognition accuracy for target micro-expression would be influenced by the context, on the basis of previous findings of context influence on facial expression recognition and affective priming [15], [20].

### Methods

#### Ethics statement

All the experimental procedures in our study were approved by the IRB of the Institute of Psychology, Chinese Academy of Sciences. All participants provided written, informed consent before taking part in our experiments. Moreover, the individual in Figure 1 has given written informed consent (as outlined in PLOS consent form) to publish his case details.

#### Participants

Thirty university students (age: 21.97±1.75 yrs, 15 females) participated for monetary remuneration. All the participants were right-handed and had normal or corrected-to-normal vision. All participants reported no history of mental illness or serious physical injuries.

#### Stimuli

One hundred and twenty-eight images of 16 models with basic facial expressions of anger, disgust, fear, happiness with mouth closed, happiness with mouth opened, sadness, surprise, and neutral were chosen from the MUG face database [26]. Taking into account the potential difficulty for people to judge facial expressions of cultural outgroup members [27], we first run an expression recognition test to select the most easily recognizable facial expressions. Thirty subjects voluntarily took part in the test, during which an expression was presented for 2000 ms and they were asked to report the expression in each image. To select materials as target expressions, subjects were asked to report by choosing one from five emotion terms (anger, disgust, fear, happiness, and surprise) in the first session of the test. To select material as context expressions, subjects were asked to report by choosing one from three emotion terms (sadness, happiness, and neutral) in the second session of the test. With a criterion of mean accuracy rate more than 80%, 48 images of 6 models (3 females, 3 males) were selected as the experiment materials. Among them, the images of happiness with mouth closed, sadness, and neutral expressions were used as positive, negative, and neutral contexts, respectively. The remaining images (anger, disgust, fear, happiness with mouth opened, and surprise) were used as the target micro-expressions. All of the images were displayed on a uniform silver-gray background in the center of the screen with a visual angle of 13.4°×13.4°.

#### Procedure

Stimuli were presented at the center of a 17-inch cathode-ray tube (CRT) monitor (frequency 100 Hz, resolution 1024×768) with the E-Prime 2.0 software package. On each trial, a black fixation cross was first presented for 500 ms in the center of the screen, followed by either a happy (with mouth closed), sad, or neutral expression context for 2000 ms. Then, one of the five target micro-expressions (anger, disgust, fear, surprise, or happiness with mouth opened) was presented for 200 ms. After that, the same context was presented for 2000 ms again. Finally, the labels of the five target expressions (anger, happiness, fear, disgust, and surprise) were presented. Participants were asked to indicate the fleeting expression by clicking one of the five labels with the mouse. The locations of the five labels on each trial were randomly presented. There were three types of contexts for each of the five target expressions of 6 models, for a total of 90 different trials. The 90 different trials were repeated three times, for a total of 270 trials. All of the trials were randomly presented. There was at least a 1-minute break after a block of 90 trials.

### Results and Discussion

**Figure 2:**
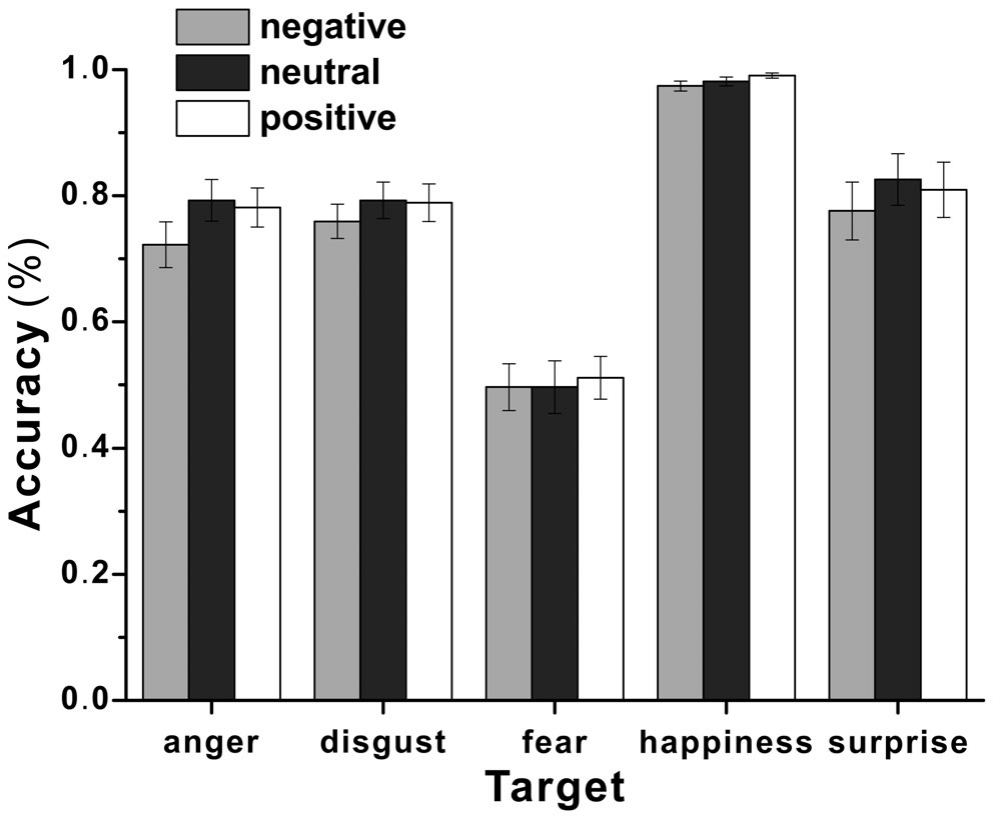
The mean accuracy rates for each target micro-expression with different contexts in Experiment 1.

Figure 2 shows the accuracy rates for each target micro-expression with three contexts. To examine whether there was an effect of emotional context on micro-expression recognition, a two-way repeated ANOVA with the context and target micro-expressions as the within-subject variables was used. A significant effect of context, *F*(2, 58) = 9.18, *p*<.001, *η_p_^2^* = .24 was revealed. Further analysis revealed that the accuracy rate was significantly lower with negative context than with neutral context, *t*(29) = −3.93, *p*<.001, *dz* = −.73, or with positive context, *t*(29) = −3.21, *p*<.01, *dz* = −.60, but no difference was found between positive and neutral contexts, *t*(29) = −.13, *p* = .85. The main effect of target micro-expression also reached significance, *F*(4, 26) = 74.31, *p*<.001, *η_p_^2^* = .92. However, the interaction of context and target micro-expressions was not significant, *F*(8, 22) = 1.19, *p* = .35. That is, the emotional context effect was not modulated by the target micro-expression.

The results demonstrated an effect of emotional context on micro-expression integration. That is, recognition of the target micro-expression was overall lower with negative (sad) context than with neutral or positive (happy) contexts. It seems like the effect was stronger on anger than others. Low recognition of the target micro-expression with negative context might have been because there were three types of negative target micro-expressions amounting to 60% of the target micro-expressions, whereas there was only one positive and one neutral target micro-expression, each amounting to only 20% of the total. Thus, to examine this possibility, we chose only anger, happiness, and neutral as the target micro-expressions in Experiment 2.

## Experiment 2

To explore whether the context effect was limited to specific materials, the stimuli used in Experiment 2 were selected from NimStim [28] instead of the MUG face database. To account for the disproportion of negative vs. positive expression, only three expressions (anger, happiness, and neutral; all with mouth opened) were selected as the target micro-expressions. Images of the three expressions anger, happiness, and neutral (all with mouth closed) were selected as the contexts. In addition, to investigate whether the context effect was influenced by the duration of the micro-expression, the presentation durations were reduced to 40 ms, 60 ms, and 80 ms in Experiment 2 on the basis of previous research [12]. We predicted that the effect of emotional context would still be observed on the basis of previous findings [20], [21]. Moreover, this effect might be modulated by the duration of the target micro-expressions.

### Methods

#### Participants

Ninety university students (age: 22.3±1.95 yrs, 45 females) participated for monetary remuneration. All the participants were right-handed and had normal or corrected-to-normal vision. All participants reported no history of mental illness or serious physical injuries. Participants were randomly assigned to three groups, thirty subjects (15 females) in each group. None of them had participated in the previous experiment.

#### Stimuli

Two hundred and ten images of 35 models (16 females, 19 males) with three types of basic facial expressions (mouth closed: anger, neutral, and happiness; mouth opened: anger, neutral, and happiness) were chosen from the NimStim database [28]. To ensure most Chinese subjects could recognize the facial expressions, we first run an expression recognition test to select the most easily recognizable facial expressions. Thirty subjects voluntarily took part in the test, during which an expression was presented for 2000 ms and they were asked to report the expression in each image. To select material as target expressions and context expressions, subjects were asked to respond by clicking one of the emotional labels (happiness, anger, or neutral) with the mouse. One hundred and twenty images of 20 models (10 females, 10 males) were selected as the experiment materials, with the criterion of mean accuracy rate more than 85%. The closed mouthed versions of the expressions for anger, happiness and neutral were used as contexts. The remaining images (facial expressions with mouth opened: anger, happiness, and neutral) were used as the target micro-expressions. All of the images were displayed on a uniform silver-gray background in the center of the screen with a visual angle of 11.8°×15.1°.

#### Procedure

The experimental procedures in Experiment 2 were identical to those in Experiment 1, except that the target micro-expression was presented for 40 ms, 60 ms, or 80 ms in each trial and participants were asked to indicate the fleeting expression by clicking one of the three labels (anger, happiness, or neutral). There were three types of contexts for each of the three target expressions of the 20 models, for a total of 180 different trials. Participants in each group were examined in only one of the three presentation durations. There was at least a 1-minute break after a block of 60 trials.

### Results and Discussion

**Figure 3:**
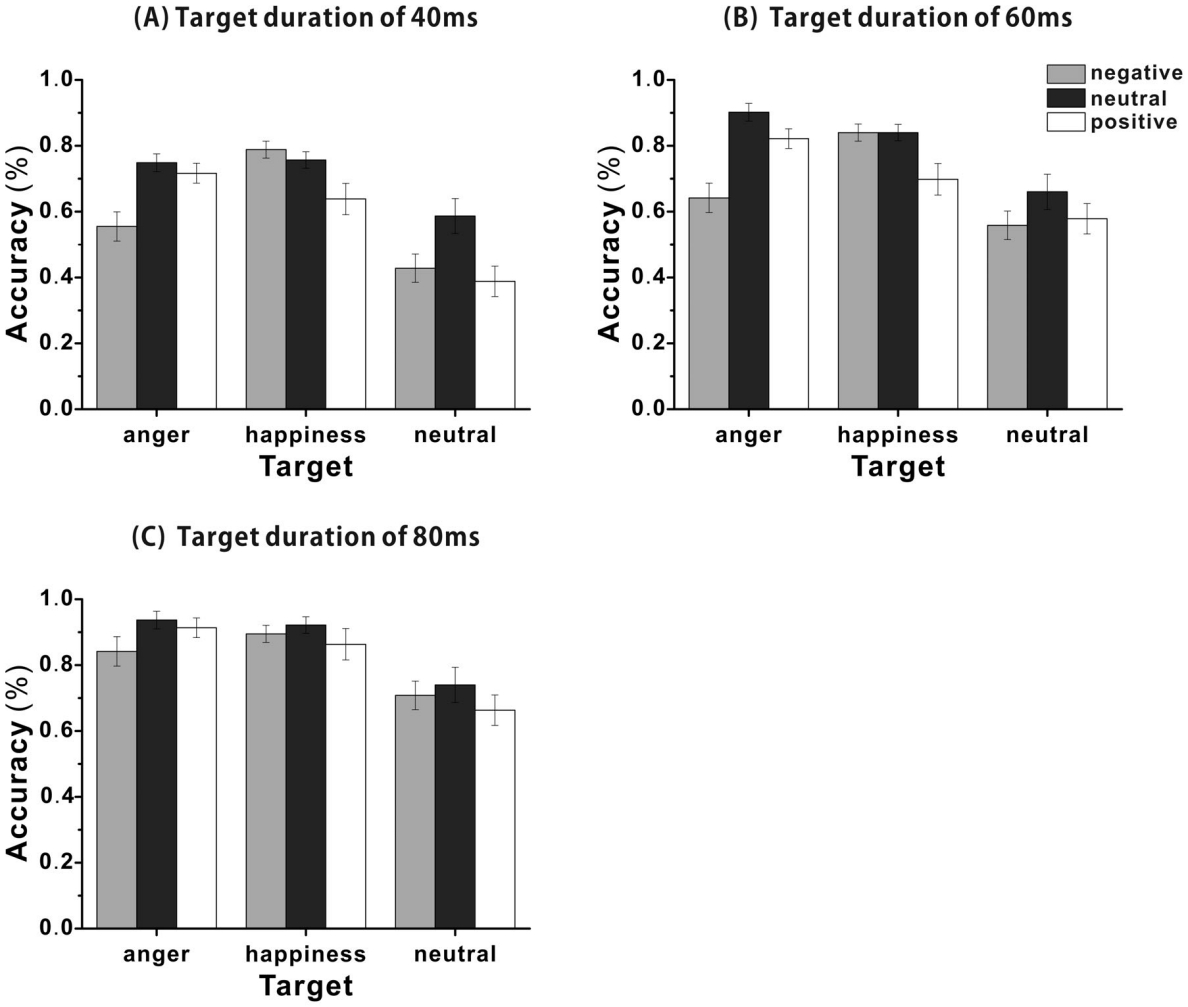
The mean accuracy rates for each target micro-expressions with different contexts in Experiment 2. (A) The mean accuracy rates for the 40 ms presentation group. (B) The mean accuracy rates for the 60 ms presentation group. (C) The mean accuracy rates for the 80 ms presentation group.

The mean accuracy rates for each micro-expression with different contexts for each presentation duration group is shown below (see Figure 3). To explore the effect of emotional context on micro-expression recognition, a three-way mixed ANOVA with the context and target micro-expressions as the within-subject variables and presentation duration as a between-subject variable was used. It revealed a significant effect of context, *F*(2, 86) = 41.09, *p*<.001, *η_p_^2^* = .49, a significant effect of target micro-expression, *F*(2, 86) = 45.87, *p*<.001, *η_p_^2^* = .52, and a significant interaction of context and target micro-expressions, *F*(4, 84) = 17.22, *p*<.001, *η_p_^2^* = .45. Further analysis revealed that the accuracy rate for anger was significantly lower with negative context than with positive or neutral context, *t*(89) = −4.76, *p*<.001, *dz* = −.50; *t*(89) = −7.96, *p*<.001, *dz* = −.84; the accuracy rate for happiness was significantly lower with positive context than with negative or neutral context, *t*(89) = −3.48, *p*<.01, *dz* = −.37; *t*(89) = −3.79, *p*<.01, *dz* = −.40; the accuracy rate for neutral was significantly higher with neutral context than with negative or positive context, *t*(89) = 3.34, *p*<.01, *dz* = .35; *t*(89) = 3.61, *p*<.01, *dz* = .38.

There was a significant main effect of presentation duration, *F*(2, 87) = 22.16, *p*<.001, *η_p_^2^* = .34, and the interaction of context and presentation duration was also significant, *F*(4, 174) = 2.81, *p*<.05, *η_p_^2^* = .06. Further analysis revealed that for each duration condition, the accuracy rate was significantly lower with negative context than with neutral context, *t*(58) = −5.94, *p*<.001, *dz* = −.78; *t*(58) = −6.72, *p*<.001, *dz* = −.88; *t*(58) = −2.83, *p*<.05, *dz* = −.37, but higher with neutral context than with positive context, *t*(58) = 5.8, *p*<.001, *d* = .76; *t*(58) = 5.05, *p*<.001, *d* = .66; *t*(58) = 2.65, *p*<.05, *d* = .34. However, no difference was observed between the accuracy rates of negative context and positive context (all *ps* >0.05).

The three-way interaction was also significant, *F*(8, 170) = 2.57, *p*<.05, *η_p_* ^2^ = .11. Further analysis revealed that the accuracy rate for anger was significantly lower with negative context than with neutral or positive context for duration conditions 40 ms and 60 ms (all *p*s <.01). The accuracy rate of happiness was significantly higher with negative context than with positive context for duration conditions 40 ms and 60 ms, *t*(29) = 2.83, *p*<.05, *d* = .53; *t*(29) = 2.68, *p*<.05, *d* = .50.

As in Experiment 1, we also found that emotional context influenced the recognition accuracy of the target micro-expressions. The results confirmed that the context effect on micro-expression recognition was not attributable to the greater number of negative target expressions in Experiment 1. In addition, in contrast to Experiment 1, we found that the effect of emotional context on recognition performance was modulated by the type of target micro-expression. The accuracy rates were lower when the valences of target micro-expression and context were consistent than when they were inconsistent. Considering that consistent expressions differed only in the mouth region (closed vs. opened), whereas the difference between the inconsistent expressions was not only in the mouth but also in the other parts of the face, one may argue that the context effect might have mainly resulted from the stimuli differences between the context and target micro-expressions, rather than from the emotional valence differences between the contexts. To test this possibility, we adopted morphed facial expressions as target micro-expressions to manipulate the stimulus discrepancy between the context and target expressions in Experiment 3.

## Experiment 3

Although it was observed that micro-expression recognition was influenced by emotional context in both Experiments 1 and 2, it remains unclear why the context effect occurred. The results of Experiment 2 indicated that the context effect might have resulted from the differences between the context and target expressions. However, previous research on facial expression recognition found that emotional valence of context could influence recognition performance [15]–[17], thus the emotional valence of context might also contribute to the context effect. In order to examine this possibility, in Experiment 3 we used morphed facial expressions as target micro-expressions that could be considered anger, happiness, or anger plus happiness. If the recognition performance had been purely based on the differences between the context and target micro-expressions, the response proportions would vary as the stimulus differences between them. Especially, when the similarity between the target expression and negative context and between the target expression and positive context was both 50%, there would be no difference on the response rates for the target micro-expressions with the two different contexts.

### Methods

#### Participants

Thirty university students (age: 22.47±1.66 years, 15 females) participated for monetary remuneration. All the participants were right-handed with normal or corrected-to-normal vision. All participants reported no history of mental illness or serious physical injuries. None of them had participated in any of the previous experiments.

#### Stimuli

The stimuli were identical to those used in Experiment 2 except that the target micro-expressions were morphed expressions. The morphed expressions were created using forty target micro-expressions (open-mouthed anger and happiness) images of 20 models in Experiment 2. We used FantaMorph 5.8 (http://www.fantamorph.com) to generate three groups of morphed facial expressions based on the image’s similarity to happiness and anger: expressions with a morph ratio of 75% happiness plus 25% anger (75% happiness), expressions with a morph ratio of 50% happiness plus 50% anger (50% happiness), and expressions with a morph ratio of 25% happiness plus 75% anger (25% happiness). We asked thirty subjects voluntarily to recognize the expression in each picture by selecting one of the three emotion labels (happiness, anger, or happiness plus anger) and to evaluate the intensity of each expression by rating the similarity of a morphed expression to the selected emotional label (ranging from 0% to 100%). Eight images with a morph proportion of 75% happiness and 25% anger, a choice rate of 90%–100% for happiness, and happiness intensity between 65%–80% were selected. Eight images with a morph proportion of 50% happiness and 50% anger, a choice rate of 47%–60% for happiness plus anger, and anger intensity between 45%–55% were selected. Eight images with a morph proportion of 25% happiness and 75% anger, a choice rate of 80%–100% for anger, and anger intensity between 65%–80% were selected. In total, 24 images of 8 models (4 females, 4 males) were selected from the forty images according to the response proportions and the intensity ratings. All of the images were displayed on a uniform silver-gray background in the center of the screen with a visual angle of 10°×12.9°.

#### Procedure

The experimental procedures were identical to those in Experiment 1, except that the target micro-expression was morphed expressions and participants were asked to indicate the fleeting expression by clicking one of the three labels (happiness, anger, and happiness plus anger) on each trial. There were three types of emotional contexts for each of the three target expressions of the 8 models, for a total of 72 different trials. The 72 different trials were repeated three times, for a total of 216 trials. All of the trials were randomly presented. There was at least a 1-minute break after a block of 72 trials.

### Results and Discussion

The response proportions of anger, happiness, and anger plus happiness for each morphed target micro-expression are shown in Table 1. To examine the influence of the emotional valence of context on micro-expression recognition, the happiness and anger response proportions were first analyzed separately and then the response proportions of happiness and anger were analyzed for the target micro-expressions with 50% happiness.

**Table 1:** The Mean Response Proportions of Morphed Micro-expressions in Experiment 3.

Target micro-expressions	Response proportions		
	Happiness	Happiness+Anger	Anger
75% happiness +25% anger	***0.74***	0.22	0.04
50% happiness +50% anger	0.18	***0.44***	0.39
25% happiness +75% anger	0.02	0.19	***0.79***

The bold italic number along the diagonal line is the main response proportions for morphed images.

**Figure 4:**
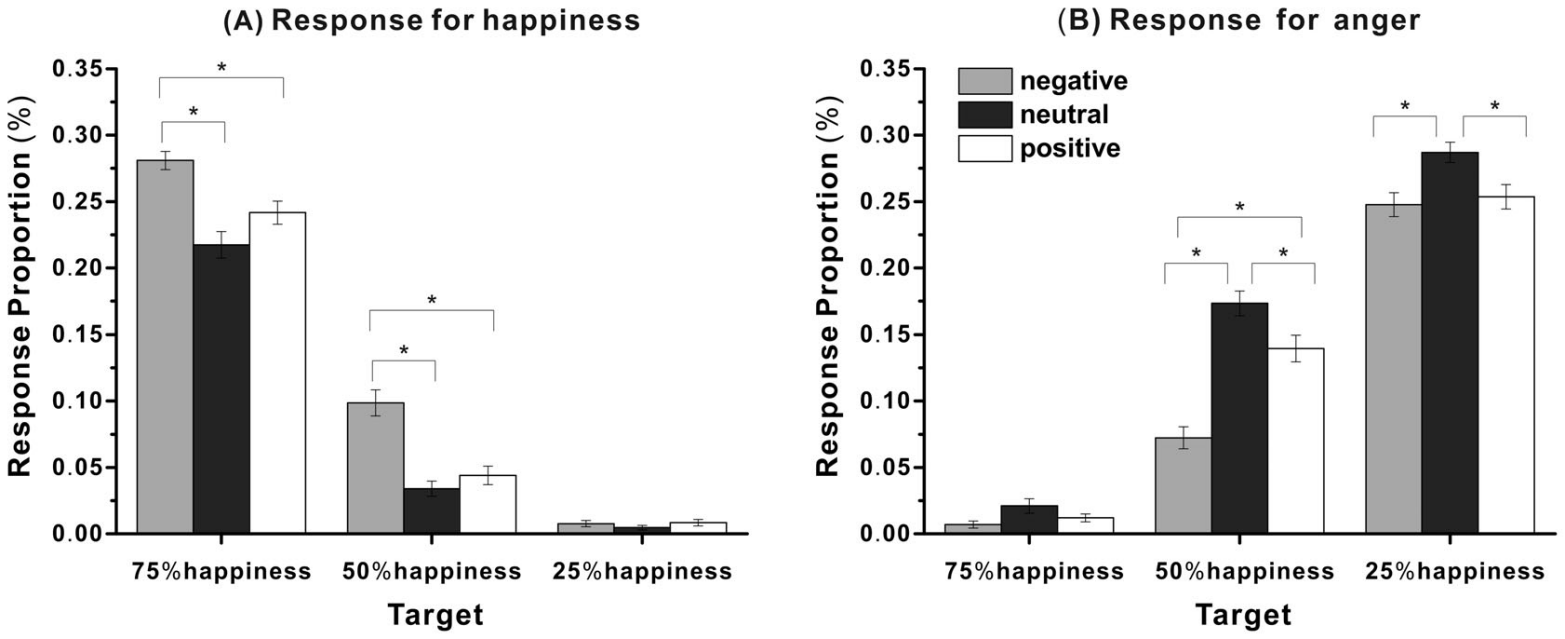
The mean response proportions for happiness and anger for each target micro-expression in Experiment 3. (A) The mean response proportions for happiness. (B) The mean response proportions for anger. * *p*<.01.

First, a repeated ANOVA on happiness response proportions with context (negative, neutral, positive) and target micro-expression (75% happiness, 50% happiness, 25% happiness) as within-subject variables was performed. It revealed a significant effect of context, *F*(2, 28) = 71.62, *p*<.001, *η_p_^2^* = .84, a significant effect of target micro-expression, *F*(2, 28) = 72.52, *p*<.001, *η_p_^2^* = .97, and a significant interaction of context and target micro-expression, *F*(4, 26) = 24.63, *p*<.001, *η_p_^2^* = .79. Further analysis showed that when the target micro-expression was 50% happiness, the happiness response proportion was significantly higher with negative contexts than with neutral or positive contexts, *t*(29) = 4.64, *p*<.001, *dz* = .86; *t*(29) = 4.23, *p*<.01, *dz* = .79. That is, even when the target micro-expression had 50% similarity to both negative and positive contexts, people recognize the target as happiness more frequently with negative contexts than with positive contexts (see Figure 4A), suggesting that the emotional valence of context influenced the micro-expression recognition.

Second, a comparable ANOVA on the anger response proportions revealed a significant effect of emotional context, *F*(2, 28) = 46.67, *p*<.001, *η_p_^2^* = .77, a significant effect of target micro-expression, *F*(2, 28) = 62.53, *p*<.001, *η_p_^2^* = .97, and a significant interaction of context and target micro-expressions, *F*(4, 26) = 16.41, *p*<.001, *η_p_^2^* = .72. Further analysis showed that when the target micro-expression was 50% happiness, the anger response proportion was significantly lower with negative context than with neutral or positive contexts, *t*(29) = −3.9, *p*<.001, *dz* = −.72; *t*(29) = −4.79, *p*<.001, *dz* = −.89, and significantly higher with neutral context than with positive context, *t*(29) = 3.78, *p*<.01, *dz* = .70. That is, even when the target micro-expression had 50% similarity to both negative and positive contexts, people recognize the target as anger more frequently with positive context than with negative context (see Figure 4B), confirming that the context effect can result from the emotional valence of context.

Finally, to examine whether emotional context influenced the response proportions of happiness and anger on the target with a morph ratio of 50% happiness plus 50% anger, a two-way ANOVA with context (negative, neutral, positive) and response category (happiness, anger) as within-subject variables was performed. It revealed a significant effect of context, *F*(2, 21) = 7.85, *p*<.01, *η_p_^2^* = .43, a significant effect of response category, *F*(1, 22) = 38.63, *p*<.001, *η_p_^2^* = .64, and a significant interaction of context and response category, *F*(2, 21) = 49.19, *p*<.001, *η_p_^2^* = .82. Further analysis revealed that the happiness response proportions were significantly lower than the anger response proportions with both neutral and positive contexts, *t*(29) = −5.04, *p*<.001, *d*z = −.94; *t*(29) = −4.63, *p*<.001, *dz* = −.85 but there was no significant difference with negative context, *t*(29) = −.53, *p* = .622.

As seen from the above results, although the similarity between the target micro-expressions and the negative (angry) or the positive (happy) context was controlled, we still found that the recognition accuracy of the target micro-expression was influenced by emotional context, as in Experiments 1 and 2. The results suggest that the emotional valence of context influenced micro-expression recognition. In particular, participants recognize the targets differently with negative and positive contexts when there was 50% similarity between target and context, regardless of whether the target was negative or positive, indicating that the context effect might have resulted from the emotional valence of context. Interestingly, when the target was 50% happiness with neutral contexts, participants gave many more anger responses than happiness responses, suggesting that negative stimuli can attract more attention than can positive stimuli.

## General Discussion

The results of Experiments 1 and 2 showed that negative context impaired micro-expression recognition regardless of the duration of the target micro-expression. In addition, the context effect on micro-expression recognition could have resulted from the stimulus differences between the context and target micro-expressions. The results of Experiment 3 showed that there was still a context effect on micro-expression recognition even when the stimulus similarity between the context and target micro-expressions was controlled. Therefore, our results suggest that the context effect on micro-expression recognition might be attributable to both the stimulus and valence differences.

Our findings are consistent with previous findings that facial expression recognition is influenced by emotional context [29], [30]. More notably, lower micro-expression recognition accuracy was observed in negative context condition than positive or neutral context conditions. This might be due to the negative context appearing before the micro-expression capturing more attention [31], [32]. Previous research showed that attention allocation is related to the emotional valence of stimuli [33] and more attentional resources are directed to negative facial expressions even though the emotional expressions are irrelevant to the task [34]. The results in Experiment 3 also showed that participants recognized the target micro-expressions with a morph ratio of 50% happiness plus 50% anger as anger more frequently than they did as happiness, confirming that negative context can attract more attention than can positive context. In addition, the individual’s expectation stemming from emotional context may also interfere with judgment of the target [35].

We found that micro-expressions were better recognized when the emotional valences of context and target were inconsistent, that is, anger was easier to recognize with positive context, whereas happiness was easier to recognize with negative context. However, previous research found that happy faces were recognized more accurately when primed by a happy face than by an angry face, whereas sad expressions were recognized more accurately when primed by an angry face than by a happy face [20]. Similar results were also observed when the facial expressions were primed by affective scenes [21]. These seemingly contradictory findings might be due to the different presentation duration of prime and target. The prime was displayed for a relatively shorter duration than were the target faces in previous studies [20], whereas the context expression was displayed much longer than was the target micro-expression in our study. Hence, it is likely that the briefly flashed prime facilitated recognition of a similar target facial expression, whereas the longer presentation of the context facial expression impaired the recognition of the similar target because of the smaller changes between the context and target expressions.

Moreover, the lower accuracy rates for inconsistent than for consistent valences trials might have been owing to the differences between stimuli. Consistent context expressions differed from target expressions only in the mouth region (closed vs. opened), whereas the differences between the inconsistent context and target expressions were in both the mouth regions and the other parts of the face. That is, the stimulus differences between the context and target micro-expressions might have led to the context effect. Previous studies have shown that the target was more easily recognized when the differences between the targets and non-targets were obvious [36]. However, it is important to note that when the target was a micro-expression with a morph ratio of 50% happiness plus 50% anger, negative context led to more happiness responses and fewer anger responses than did neutral context, whereas positive context led to more anger responses than did neutral context. These results revealed that the valence differences between contexts also contributed to the effect of emotional context. Therefore, the context effect on micro-expression recognition might be owing to not only the stimulus differences between the context and target micro-expressions but also the valence differences between contexts.

Previous research has shown that the processes of facial expression recognition are not simple classification but are cognitive processes including the results of sequential and cumulative stimulus evaluations that took the context information into account [37]. However, it remains unclear exactly how emotional context influences micro-expression recognition. The current study has provided behavioral evidence for the role of emotional context information in micro-expression recognition. Further studies should use neuroimaging techniques to reveal the stages in micro-expression processing that are influenced by the emotional context.

In summary, the present study provides evidence that emotional context influences micro-expression recognition. The context effect on micro-expression recognition might be attributable to both the stimulus and valence differences.
